# UiO-66-(COOH)_2_ Decorated Collagen Fiber Membranes for High-Efficiency Separation of Cationic Surfactant-Stabilized Oil/Water Emulsions: Toward Sustainable and Robust Wastewater Treatment

**DOI:** 10.3390/polym17212879

**Published:** 2025-10-29

**Authors:** Guifang Yang, Qiu Wu, Gao Xiao, Xiaoxia Ye

**Affiliations:** 1 Fujian Provincial Key Laboratory of Ecological Impacts and Treatment Technologies for Emerging Contaminants, College of Environmental and Biological Engineering, Putian University, Putian 351100, China; abc396550322@ptu.edu.cn; 2 Key Laboratory of Ecological Environment and Information Atlas, Putian University, Putian 351100, China; 3 College of Environment and Safety Engineering, Fuzhou University, Fuzhou 350108, China; 230620061@fzu.edu.cn

**Keywords:** collagen fiber membrane, cationic emulsion demulsification, surfactant, charge neutralization, size exclusion

## Abstract

Membrane separation is a promising technology for emulsified wastewater treatment. However, conventional membrane often suffer from limitations such as low mechanical strength, the inherent “trade-off” effect between flux and separation efficiency, and poor antifouling properties. To address these challenges, we report a novel composite membrane (CFM-UiO-66-(COOH)_2_) fabricated by in situ growth of functionalized UiO-66-(COOH)_2_ on a mechanically robust collagen fiber membrane (CFM) substrate. The resulting composite leverages the inherent properties of the CFM, along with the controlled generation of charge-neutralization demulsification sites and size-sieving filtration layers from the UiO-66-(COOH)_2_. This CFM-UiO-66-(COOH)_2_ exhibited superwetting behavior and achieved efficient separation of cationic surfactant-stabilized oil-in-water micro- and nano-emulsions. Specifically, the CFM-UiO-66-(COOH)_2_ achieved separation efficiencies exceeding 99.85% for various cationic O/W emulsions, with permeation fluxes ranging from 178.9 to 225.9 L·m^−2^·h^−1^. The membrane also demonstrated robust antifouling properties, excellent acid/alkali resistance, high abrasion durability, and good biocompatibility. Importantly, stable performance was maintained over six consecutive separation cycles. These characteristics, combined with the electrostatic interactions between carboxyl groups on the UiO-66-(COOH)_2_ and cationic contaminants, suggest that CFM-UiO-66-(COOH)_2_ holds significant potential for practical and sustainable wastewater treatment applications.

## 1. Introduction

The increasing discharge of oily wastewater from industries such as metal smelting [1], food processing, leather tanning, and petrochemical production [2], compounded by marine oil spillage from transportation, has elevated oily wastewater pollution as a critical environmental challenge [3]. The excessive use of cationic surfactants in industrial and domestic applications is of particular concern. These surfactants, typically composed of positively charged, nitrogen-containing groups with amphiphilic properties, readily stabilize oil-in-water (O/W) emulsions upon entering aquatic systems, posing significant threats to ecosystems and human health [4]. Traditional separation methods, including flotation, centrifugation, gravity separation, coagulation, adsorption, and biological treatment, have historically been employed for emulsion treatment [5]. However, their inherent drawbacks, such as high energy consumption, elevated costs, inefficiency, and the risk of secondary pollution, have become increasingly apparent with the advent of advanced separation technologies. Membrane separation technology has gained prominence due to its operational simplicity, low energy demand, high efficiency, and mild processing conditions. Nevertheless, membrane separation technology still faces critical challenges in oily wastewater treatment, including inadequate mechanical robustness, poor reusability, and the persistent flux-selectivity trade-off [6]. Therefore, improving the mechanical strength, recyclability, separation flux, and efficiency of membrane separation materials is the key to effectively separating emulsion wastewater.

Waste valorization has emerged as a cornerstone of sustainable production, with the “waste to resource” strategy offering a direct and effective pathway [7]. However, vast quantities of waste, such as collagen-rich leather byproducts (where less than 35% of raw hides are utilized) [8], remain underutilized, contributing to resource waste and secondary pollution [9,10]. As a material derived from animal skin, collagen fibrous membranes (CFM) constitute both an abundant and a renewable biomass resource. CFM, formed by self-assembly of type I collagen molecules, possesses a unique hierarchical structure. This structure ranges from nanoscale protofibrils (10–500 nm) to micrometer-sized fiber bundles, forming a three-dimensional fibrous network [1]. This architecture endows CFM with excellent mechanical properties, including a tensile strength of up to 38 MPa and a tear strength of 46 N·mm^−1^ [11]. Moreover, the micro- and nano-composite structure generates a capillary effect that significantly reduces the resistance to fluid transport [12]. Furthermore, collagen fibers possess an amphiphilic nature arising from the coexistence of hydrophilic domains, contributed by polar amino acids, and hydrophobic domains, conferred by non-polar amino acids. This amphiphilicity facilitates both covalent and non-covalent modifications, thereby enabling the enhancement of their separation performance. The abundant side chain functional groups (-COOH, -NH_2_, -OH, etc.) also render CFM highly chemically reactive, enabling both coordination with metal ions [13,14,15] and modulation of the surface charge to facilitate electrostatic destabilization of emulsions. This integration of fiber architecture, mechanical robustness, capillarity, and chemical activity paves the way for novel, eco-friendly emulsion separation materials.

Current membrane technologies for emulsion separation primarily rely on size sieving and electrostatic interactions. While CFM possesses multiscale pore structures (1 nm–200 μm) [16], their broad pore size distribution limits separation precision. Meanwhile, leather collagen fiber membranes lack specific functional sites, which severely limit their application in specific environments. Therefore, most studies in the literature focus on regulating their active sites [17,18,19]. Metal–organic frameworks (MOFs), composed of inorganic metal nodes and organic ligands, offer a compelling solution. Their tunable pore architectures, high surface areas, and compatibility with diverse substrates have enabled breakthroughs in gas storage [20], separation [21,22], catalysis [23], energy applications [24], and water treatment [25]. By tailoring organic ligands (e.g., -COOH in UiO-66-(COOH)_2_ or -NH_2_ in UiO-66-NH_2_) [26,27,28], MOFs can create sieving layers with precise pore sizes while introducing functional groups for electrostatic demulsification. However, the powder structure of MOFs makes their recovery extremely difficult, and it is challenging to ensure the stability of their performance during multiple cycles [29,30,31].

This study presents an innovative approach involving the in situ growth of UiO-66-(COOH)_2_ onto CFM, resulting in three synergistic effects. The MOFs coating creates regulated pore channels that function as a selective size-sieving layer, while the carboxyl groups of UiO-66-(COOH)_2_ enhance hydrophilicity and provide charge-neutralization demulsification sites. Furthermore, CFM’s inherent capillary action of the CFM substrate significantly improves flux. The MOFs-CFM composite demonstrates exceptional environmental durability, maintaining robust acid/alkali resistance, sustained abrasion tolerance through extended wear cycles, and stable separation efficiency during repeated reuse under harsh operational conditions. This high-performance material establishes an eco-friendly separation strategy with broad applicability for complex emulsion remediation.

## 2. Materials and Methods

### 2.1. Materials

CFM was sourced from the subdermal layer of tanned hides, which remains following the separation of the primary grain layer during leather processing. 1,2,4,5-Benzenetetracarboxylic acid (TPA), cetyltrimethylammonium bromide (CTAB), zirconyl nitrate, bromohexadecyl pyridine, and trifluoroacetic acid (TFA) were purchased from Aladdin Industrial Corporation (Shanghai, China). Acetone, n-hexadecane, n-dodecane, n-octane, and n-hexane were purchased from Sinopharm Chemical Reagent Co., Ltd. (Shanghai, China). All chemicals were of analytical grade and used as received without further purification.

### 2.2. Preparation of CFM- UiO-66-(COOH)_2_

A zirconium precursor solution was first prepared by dissolving a specified amount of zirconyl nitrate hydrate in 15 mL of ultrapure water under ultrasonication to obtain Zr^4+^ precursor solutions with varying concentrations (0, 20, 32, 44, and 56 mmol L^−1^). Pre-cut CFM were immersed in the precursor solutions and subjected to rotational shaking at room temperature for 2 h. Concurrently, the organic ligand (TPA) was dissolved in 15 mL of ultrapure water and heated at 100 °C for 10 min, maintaining a molar ratio of 2:1 (organic ligand to metal ions). After cooling to room temperature, the ligand solution was combined with the Zr^4+^ precursor mixture, followed by the addition of 5 mL TFA. The reaction proceeded under continuous rotational shaking at room temperature for 24 h. Post-synthesis, the modified membranes were ultrasonically cleaned three times with anhydrous ethanol and ultrapure water to remove unreacted residues. Subsequently, the membranes were immersed in acetone for 48 h, with the acetone replaced every 24 h. Finally, the membranes were rinsed with ethanol and ultrapure water, then freeze-dried for subsequent use.

### 2.3. Preparation of Cationic O/W Emulsions

The O/W microemulsions containing cationic surfactants were formulated as follows: ME1 was n-hexadecane in water emulsion containing CPB, ME2 was dodecane in water emulsion containing CPB, ME3 was n-octane in water emulsion containing CPB, and ME4 was n-hexane in water emulsion containing CPB. The specific formulation method was as follows: 0.01 g of CPB was dissolved in 1 L of ultrapure water and placed under a mechanical stirrer at 500 rpm for 15 min, followed by the slow addition of 10 mL of the oil phase to the above solution drop by drop and the mechanical stirring was continued at 1000 rpm for 20 min, which resulted in the O/W-type microemulsions.

The O/W nanoemulsions containing cationic surfactants were formulated as follows: NE1 was a CTAB containing water in dodecane emulsion, NE2 was a CTAB containing water in n-hexadecane emulsion, NE3 was a CTAB containing water in n-octane emulsion, and NE4 was a CTAB containing water in n-hexane emulsion. The specific preparation method was as follows: 0.01 g of CTAB was dissolved in 1 L of ultrapure water and placed under a mechanical stirrer at 500 rpm for 15 min, followed by the slow addition of 10 mL of the oil phase drop by drop to the above solution and continued mechanical stirring at 5000 rpm for 60 min, which resulted in the O/W type nanoemulsions.

### 2.4. Emulsion Separation

Emulsion separation was conducted in an H-type equipment, with the CFM-UiO-66-(COOH)_2_ fixed between the feed cell and the collecting cell. The prepared emulsion was poured into the feed cell and the corresponding filtrate was obtained in the collecting cell. The collected filtrate was measured for oil content using an infrared oil meter and the separation efficiency with separation fluxes was calculated using the following equation:

(1) Separation efficiency=100%−C

where *C* is the the percentage of oil content in the filtrate.

The equation for separation fluxes was shown as follows:

(2) $$J=\frac{V}{S\times t}$$

where *J* is the flux of the membrane, L·m^−2^·h^−1^; *V* is the filtrating volume, L; *S* is the area of the membrane, m^2^; *t* is the time used for emulsion separation, h.

### 2.5. Abrasion Resistance, Acid and Alkali Stability, Biocompatibility Evaluation of CFM-UiO-66-(COOH)_2_

To comprehensively evaluate the durability and safety of membranes, three systematic tests were conducted.

Abrasion resistance: Membranes were subjected to cyclic wear tests using 100 grit sandpaper under a constant load of 650 g·cm^2^, with each cycle defined as a 15 cm friction distance. Post-abrasion performance (contact angle, flux, and separation efficiency) was assessed after 100, 200, 300, 400, and 500 cycles.

Acid and alkali stability: Membranes were immersed in aqueous solutions (pH 2–12) for 24 h, dried at 60 °C for 6 h, and tested for NE1 nanoemulsion separation efficiency and flux.

Biocompatibility evaluation: CFM and CFM-UiO-66-(COOH)_2_ were placed in the same culture vessel with six zebrafish with 87% similarity to human genes, and a separate copy of six zebrafish was prepared as a blank group control, which were fed and cleaned from the aquatic environment every three days at intervals of five days, and their survival was recorded at intervals of five days.

### 2.6. Characterization

The surface morphologies of CFM and CFM-UiO-66-(COOH)_2_ were observed using field emission scanning electron microscopy (FE-SEM, Nova Nanosem 450, FEI, Hillsboro, OR, USA). Fourier transform infrared spectroscopy (FT-IR, Nicolet iS10, Thermo Fisher Scientific, Waltham, MA, USA) was employed to analyze the functional groups of CFM-UiO-66-(COOH)_2_ nanoparticles, CFM, and composite membranes. The crystalline phases of the materials were characterized by X-ray diffraction (XRD, MiniFlex II, Rigaku, Tokyo, Japan). Surface elemental compositions and chemical states were determined via X-ray photoelectron spectroscopy (XPS, ESCALAB 250, Thermo Fisher Scientific, MA, USA). Water contact angles were measured using a contact angle goniometer (OSA200-01, Ningbo New Boundary Instrument, Ningbo, China). Dynamic light scattering (DLS, BI-200SM, Brookhaven Instruments, Holtsville, NY, USA) was utilized to evaluate the droplet size distribution of emulsions. Specific surface areas and pore size distributions were analyzed using nitrogen adsorption–desorption measurements (ASAP 2460, Micromeritics, Norcross, GA, USA). The oil content in the filtrate was quantitatively determined with an infrared oil analyzer (OIL-460, Huaxia Create, Beijing, China).

## 3. Results and Discussion

### 3.1. Characterization of the CFM-UiO-66-(COOH)_2_

**Figure 1:**
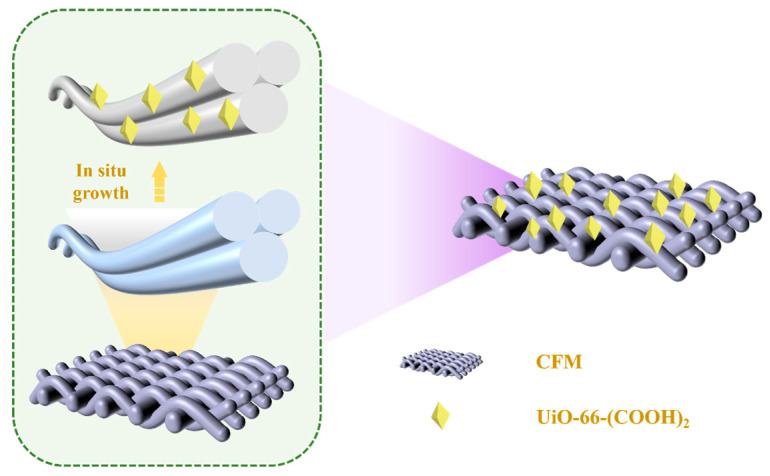
The schematic diagram of CFM-UiO-66-(COOH)_2_ preparation.

**Figure 2:**
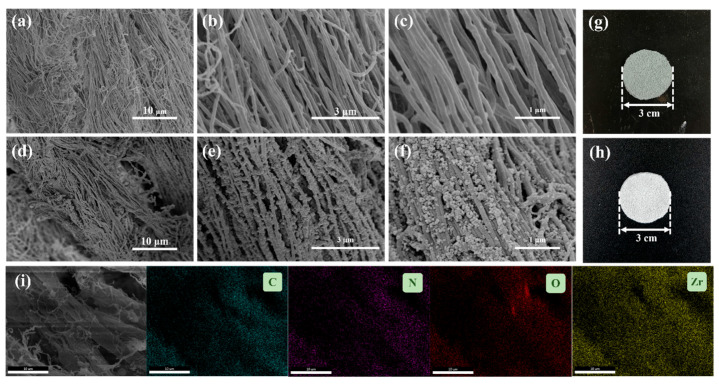
(**a**–**c**) The scanning electron micrographs of CFM, (**d**–**f**) The scanning electron micrographs of CFM-UiO-66-(COOH)_2_, (**g**) The digital photograph of CFM, (**h**) The digital photograph of CFM-UiO-66-(COOH)_2_, (**i**) The SEM-EDS mapping images of CFM-UiO-66-(COOH)_2_.

Figure 1 illustrates the fabrication process of the CFM-UiO-66-(COOH)_2_ composite membrane. To investigate the morphological changes induced by modification, scanning electron microscopy (SEM) was performed on both the pristine CFM and modified CFM-UiO-66-(COOH)_2_ composite. As shown in Figure 2a–c, the unmodified CFM exhibited a distinct nanofiber structure with clean, smooth surfaces free of any attachments. After modification with UiO-66-(COOH)_2_, the CFM retained its original nanofibrous structure, as observed morphologically. The UiO-66-(COOH)_2_ particles were observed to be uniformly distributed on the fiber surfaces, forming a hierarchical nanostructure integrated with the CFM framework (Figure 2d–f). Elemental mapping analysis of the CFM-UiO-66-(COOH)_2_ confirmed the uniform distribution of four characteristic elements (C, N, O, and Zr) across the collagen fiber surfaces (Figure 2i). The detection of Zr provided direct evidence for the successful in situ growth of UiO-66-(COOH)_2_ particles on the CFM substrate.

**Figure 3:**
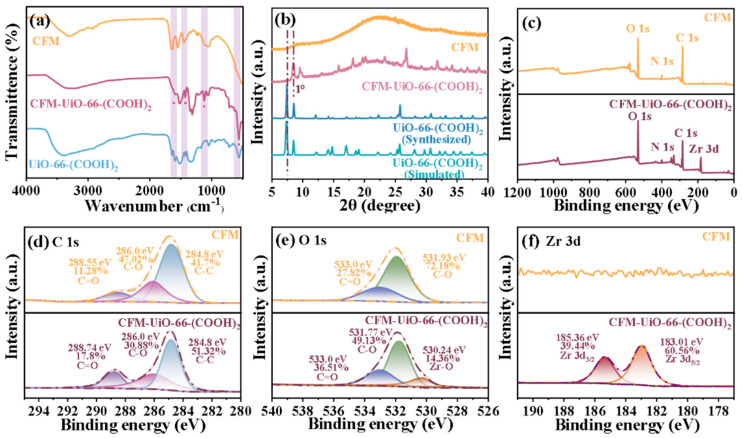
(**a**) FT−IR spectra of CFM-UiO-66-(COOH)_2_, (**b**) XRD patterns of simulated UiO-66-(COOH)_2_, as-synthesized UiO-66-(COOH)_2_, CFM and CFM-UiO-66-(COOH)_2_, (**c**–**f**) The XPS survey scan of CFM and CFM-UiO-66-(COOH)_2_.

To further analyze the chemical structural changes in CFM-UiO-66-(COOH)_2_ and verify the successful growth of nanoparticles, FT-IR spectroscopy was performed on pristine CFM, UiO-66-(COOH)_2_, and the synthesized CFM-UiO-66-(COOH)_2_. As shown in Figure 3a, the spectrum of UiO-66-(COOH)_2_ exhibited a characteristic peak at 1705 cm^−1^, corresponding to the C=O stretching vibration of uncoordinated carboxyl groups (-COOH) in the UiO-66-(COOH)_2_ linkers. The presence of this peak not only confirmed the successful growth of UiO-66-(COOH)_2_ on the CFM matrix but also indicated the availability of free carboxyl groups within the composite material for subsequent applications. A peak at 1502 cm^−1^, attributed to the aromatic C=C stretching vibration from the UiO-66-(COOH)_2_ organic ligands, was observed in both the UiO-66-(COOH)_2_ powder and UiO-66-(COOH)_2_ spectra, but was absent in pristine CFM, providing definitive evidence for the successful in situ growth of the MOFs on the CFM substrate. The characteristic peak at 1580 cm^−1^ corresponded to the O-C-O symmetric stretching vibration, associated with the coordination between Zr and carboxylate groups. Additionally, the peak observed at 1200 cm^−1^ was likely due to C-O-H bending vibrations of the carboxylic acid groups in the organic linkers. The sharp characteristic peak at 654 cm^−1^ in the CFM-UiO-66-(COOH)_2_ corresponded to the Zr-O stretching vibration of UiO-66-(COOH)_2_. These collective results confirmed the effective chemical modification of the CFM substrate via in situ growth of UiO-66-(COOH)_2_, while maintaining the structural integrity of the membrane.

Further confirmation of the successful integration of crystalline CFM-UiO-66-(COOH)_2_ was obtained through XRD analysis of the CFM-UiO-66-(COOH)_2_. As shown in Figure 3b, the XRD pattern of UiO-66-(COOH)_2_ modified collagen fiber membrane exhibited nearly identical peak positions and intensities compared to pure UiO-66-(COOH)_2_, with only minor peak shifts (∼1°). These slight deviations likely resulted from lattice defects induced by the constrained growth of UiO-66-(COOH)_2_ crystals within the collagen fiber matrix. Notably, the CFM-UiO-66-(COOH)_2_ retained the characteristic strong diffraction peaks of pristine CFM at 7° and 20°, confirming preservation of collagen’s native triple helical structure following UiO-66-(COOH)_2_ incorporation. This demonstrated that the in situ growth process facilitated the successful infiltration of CFM-UiO-66-(COOH)_2_ nanoparticles into the collagen fiber network without disrupting the underlying crystalline domains of the CFM.

To precisely characterize the interfacial interactions between UiO-66-(COOH)_2_ and CFM, and to further investigate changes in elemental binding states upon CFM-UiO-66-(COOH)_2_ incorporation, XPS was performed on the CFM-UiO-66-(COOH)_2_. As shown in Figure 3c, comparison of the XPS survey spectra of CFM and CFM-UiO-66-(COOH)_2_ revealed the presence of carbon (C 1s), nitrogen (N 1s), and oxygen (O 1s) signals in both materials. However, only the CFM-UiO-66-(COOH)_2_ composite exhibited detectable zirconium (Zr 3d) signals. From the high resolution C 1s spectra, the deconvolved peaks at 288.74 eV, 286.0 eV, and 284.8 eV corresponded to C=O, C-O, and C-C bonds, respectively (Figure 3d). The observed increase in C=O content was attributed to the organic ligands and free -COOH groups in UiO-66-(COOH)_2_ within the composite, while the decrease in C-O signal suggested the interaction between Zr^4+^ and hydroxyl groups (-OH) on the CFM surface. The O 1s spectrum of CFM-UiO-66-(COOH)_2_ (Figure 3e) exhibited a higher overall intensity compared to that of unmodified CFM. The appearance of a new O 1s peak at 530.24 eV served as compelling evidence for the successful CFM modification, resulting from the coordination of Zr^4+^ metal ions with -COOH groups on collagen fibers to form Zr-O bonds. In the Zr 3d spectrum, peaks were observed at 185.36 eV and 183.01 eV, corresponding to Zr 3d_3/2_ and Zr 3d_5/2_ (Figure 3f). Notably, no Zr signal was detected in the CFM spectrum, providing further evidence for the successful incorporation of UiO-66-(COOH)_2_ into the collagen fiber matrix.

### 3.2. Separation Performance Analysis of CFM-UiO-66-(COOH)_2_ for Cationic O/W Emulsions

**Figure 4:**
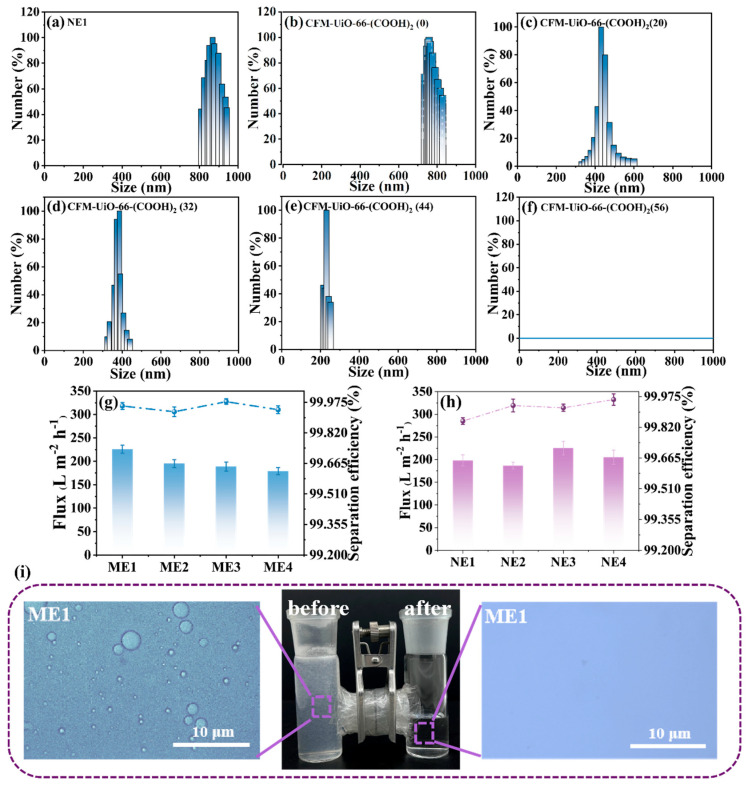
(**a**–**f**) DLS curves of NE1, after the separation of CFM-UiO-66-(COOH)_2_(x) with increased dosage of Zr^4+^ precursor from 0 mmol·L^−1^ to 20 mmol·L^−1^, 32 mmol·L^−1^, 40 mmol·L^−1^, and 56 mmol·L^−1^, (**g**,**h**) Emulsion separation flux and efficiency of ME1-ME4 and NE1-NE4, (**i**) The optical microscope images of ME1 before and after separation.

To optimize the UiO-66-(COOH)_2_ loading and its impact on membrane separation performance, a series of CFM-UiO-66-(COOH)_2_(x) were fabricated and evaluated for the separation of CTAB-stabilized NE1 emulsions. Here, ‘x’ represented the Zr^4+^ precursor concentration, with tested values of 0, 20, 32, 44, and 56 mmol·L^−1^. The resulting CFM-UiO-66-(COOH)_2_(x) were then installed in an H-type separation device for NE1 emulsion separation. The separation performance was evaluated by analyzing the particle size distribution of the filtrate using dynamic light scattering (DLS). As shown in Figure 4a, the untreated NE1 emulsion exhibited a particle size distribution ranging from 809 to 943 nm. Filtration through unmodified CFM-UiO-66-(COOH)_2_(0) resulted in only a modest reduction in particle size, with a distribution of 735 to 831 nm (Figure 4b), indicating limited separation capability. In contrast, as the Zr^4+^ precursor concentration was progressively increased up to 44 mmol·L^−1^, the particle size distribution of the filtrate showed a gradual shift toward smaller diameters, with measured distributions of 325–599 nm, 320–440 nm, and 204–263 nm for the increasing concentrations (Figure 4c–e). This progressive decrease in particle size demonstrated the enhanced filtration performance achieved with increasing CFM-UiO-66-(COOH)_2_ loading. Notably, when the Zr^4+^ precursor concentration reached 56 mmol·L^−1^, DLS analysis revealed no detectable particles in the filtrate, indicating the complete removal of emulsion droplets and surfactant molecules (Figure 4f). This result confirmed the excellent separation capability of the optimized membrane. These results clearly demonstrated that CFM-UiO-66-(COOH)_2_ facilitated efficient separation of cationic NE1 emulsions, with separation performance directly correlating to the Zr^4+^ precursor concentration used during synthesis. The XRD patterns of CFM-UiO-66-(COOH)_2_ prepared with different Zr^4+^ precursor concentrations (Figure S1) clearly demonstrated that the diffraction peak intensities characteristic of UiO-66-(COOH)_2_ increased with higher Zr^4+^ concentrations. This observation indicated that increasing the Zr^4+^ concentration enhanced the grafting amount of UiO-66-(COOH)_2_ onto the collagen fiber membrane. The improved separation performance could be attributed to greater penetration and incorporation of UiO-66-(COOH)_2_ within the collagen fiber network. The strong correlation between Zr^4+^ precursor concentration and separation performance confirmed the tunable and controllable nature of CFM-UiO-66-(COOH)_2_, enabling customized separation efficiency tailored to specific emulsion characteristics and requirements.

To evaluate the broad applicability of CFM-UiO-66-(COOH)_2_(56), its separation performance was systematically evaluated for a range of cationic surfactant-stabilized micro/nanoemulsions. As shown in Figure 4g and Figure S2, the membrane effectively separated cetylpyridinium bromide CPB stabilized O/W emulsions containing different hydrocarbon phases (n-hexadecane, n-dodecane, n-octane, and n-hexane). Stereomicroscopic images of the ME1 emulsion feed revealed the presence of oil droplets (>1 μm) and significant turbidity, indicative of a high concentration of undissolved oil. In contrast, stereomicroscopic examination of the filtrate following filtration with CFM-UiO-66-(COOH)_2_ revealed no detectable emulsion droplets, demonstrating the membrane’s effective separation of this cationic surfactant-stabilized microemulsion. Consistent separation performance was observed for ME2-ME4 emulsions, with flux rates of 225.872, 195.156, 188.675, and 178.976 L·m^−2^·h^−1,^ respectively. Notably, all systems achieved separation efficiencies exceeding 99.927%, highlighting the broad applicability of CFM-UiO-66-(COOH)_2_ for the treatment of cationic surfactant-stabilized microemulsions.

Additionally, CFM-UiO-66-(COOH)_2_ also demonstrated efficient separation of nanoemulsions stabilized by cationic surfactants (Figure 4h and Figure S3). Taking NE1 as an example, the original NE1 nanoemulsion exhibited a particle size distribution between 809 and 943 nm, as measured by DLS. After separation with CFM-UiO-66-(COOH)_2_, no detectable particle size distribution was observed in the filtrate, confirming the effectiveness of the separation. Similarly, the DLS results for NE2-NE4 filtrates also showed no measurable particle size distribution, further demonstrating the membrane-efficient separation of nanoemulsions. The separation fluxes for NE1-NE4 were 198.109, 186.581, 225.118, and 205.124 L·m^−2^·h^−1^, respectively, with separation efficiencies exceeding 99.851% in all cases. These findings demonstrated that CFM-UiO-66-(COOH)_2_ exhibited highly efficient separation capabilities for a range of surfactant-stabilized nanoemulsions (Figure 4i).

### 3.3. Antifouling Evaluation of CFM-UiO-66-(COOH)_2_

#### 3.3.1. Reusability of CFM-UiO-66-(COOH)_2_

**Figure 5:**
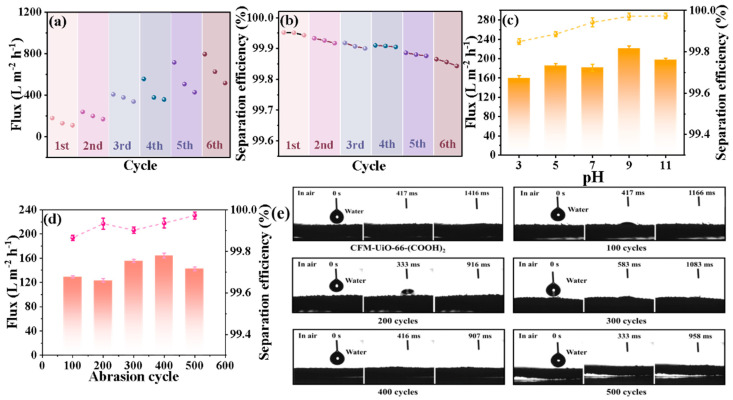
(**a**,**b**) Separation fluxes and efficiencies of NE1 obtained during the 6 cycles of separation by using the CFM-UiO-66-(COOH)_2_(56), (**c**) Separation fluxes and separation efficiencies of the CFM-UiO-66-(COOH)_2_ immersed in aqueous solutions at varied pH (3–11) for 24 h to NE1, (**d**,**e**) Separation fluxes and separation efficiencies of the CFM-UiO-66-(COOH)_2_ membrane abraded with sandpaper for different cycles to NE1 and the WCAs digital photos of the CFM-UiO-66-(COOH)_2_ membrane abraded with sandpaper for different cycles.

Reusability serves as a critical parameter for evaluating the long-term viability and antifouling properties of emulsion separation membranes. To systematically assess the antifouling performance of CFM-UiO-66-(COOH)_2_, cyclic separation tests were performed using NE1 nanoemulsion as a model system. Each separation cycle lasted for 1 h, with filtrate samples collected at 20 min intervals to monitor separation efficiency. After each separation cycle, the CFM-UiO-66-(COOH)_2_ was regenerated by ultrasonic cleaning in a 1:1 mixture of absolute ethanol and ultrapure water, followed by drying in an oven at 60 °C for 2 h to ensure complete solvent removal. This regeneration protocol was repeated for six consecutive separation cycles to evaluate the membrane’s reusability and antifouling stability. As shown in Figure 5a–b, the CFM-UiO-66-(COOH)_2_ exhibited remarkable stability over six consecutive NE1 nanoemulsion separation cycles. Notably, the separation flux increased progressively from 179.010 to 796.020 L·m^−2^·h^−1^ with cycling number, while maintaining exceptionally high separation efficiency (99.843–99.952%). This performance enhancement suggested that the unique fibrous architecture of CFM-UiO-66-(COOH)_2_ effectively mitigates membrane fouling, specifically by preventing oil droplet-induced pore blockage. The effectiveness of simple ethanol cleaning in restoring pore accessibility, leading to significantly enhanced flux in subsequent cycles, underscores the robust antifouling character of the membrane. These findings demonstrated that CFM-UiO-66-(COOH)_2_ possesses exceptional antifouling properties, offering both economic and environmental advantages through performance-stable reuse without efficiency degradation.

#### 3.3.2. Acid and Alkali Resistance Analysis of CFM-UiO-66-(COOH)_2_

In practical emulsion wastewater separation applications, membrane materials are often exposed to harsh chemical environments, including strongly acidic or alkaline conditions. Therefore, adequate chemical stability is a critical requirement for the long-term viability of separation membranes. To evaluate the acid and alkali resistance of the CFM-UiO-66-(COOH)_2_, membranes were immersed in solutions with pH values ranging from 3 to 11 (in intervals of 2 pH units) for 24 h. As shown in Figure 5c, after exposure to acidic and alkali solutions and subsequent use in separating the NE1 emulsion, the membranes maintained stable separation efficiency (99.849–99.973%) with minimal flux loss (159.203–220.981 L·m^−2^·h^−1^). These results clearly demonstrated that CFM-UiO-66-(COOH)_2_ possessed excellent resistance to both acidic and alkaline environments, retaining its separation performance even under extreme pH conditions. This remarkable stability highlighted the membrane’s exceptional durability and resilience in harsh operating environments. For industries routinely handling corrosive substances, such robust chemical resistance represents a critical practical advantage.

#### 3.3.3. Abrasion Resistance Analysis of CFM-UiO-66-(COOH)_2_

In membrane separation technology, insufficient mechanical strength can lead to performance degradation due to operational wear. To evaluate the abrasion resistance of CFM-UiO-66-(COOH)_2_, systematic wear testing was conducted using 100–500 cycles of sandpaper abrasion. Figure S4 showed the apparent morphology of CFM-UiO-66-(COOH)_2_ after different wear cycles. Figure S5 presents SEM images of CFM-UiO-66-(COOH)_2_ after 100, 200, 300, 400, and 500 sandpaper abrasion cycles, illustrating its structural changes. As shown in the figures, with increasing wear cycles, CFM-UiO-66-(COOH)_2_ underwent multiscale deformation. The abraded fiber bundles became looser, transitioning from large, entangled clusters into smaller, well-separated individual bundles. The resulting micron-scale fiber bundles and dispersed nanofibers increased the membrane’s roughness, thereby enhancing its contact area with air.

This increase in surface roughness is expected to improve hydrophilicity, as predicted by the Cassie-Baxter equation. Indeed, water contact angle measurements confirmed that the abraded CFM-UiO-66-(COOH)_2_ exhibited significantly improved hydrophilicity compared to the non-abraded membrane, with complete water droplet absorption occurring within 958 ms (Figure 5e). Notably, when applied to NE1 emulsion separation, the abraded membranes maintained outstanding separation performance without observable degradation in flux or efficiency. The separation flux remained stable between 123.380 and 164.183 L·m^−2^·h^−1^, while separation efficiency consistently exceeded 99.865% (Figure 5d), indicating that the wear treatment did not compromise the membrane’s emulsion separation capability. These results demonstrated that CFM-UiO-66-(COOH)_2_ maintained its high separation efficiency even after significant mechanical abrasion, indicating exceptional durability and mechanical stability. The sustained performance under abrasive conditions highlights the potential for long-term application in demanding separation processes.

#### 3.3.4. Biosafety Evaluation of CFM-UiO-66-(COOH)_2_

Given the increasing concern regarding the environmental impact of materials, it is crucial to evaluate the potential biosafety of the CFM-UiO-66-(COOH)_2_, particularly as it is intended for emulsion separation in water treatment applications. To evaluate the biosafety of CFM-UiO-66-(COOH)_2_, zebrafish (Danio rerio), a widely used model organism with high genetic similarity to humans (∼87%), were exposed to the material. Eighteen zebrafish were divided into three groups and cultured in pure water, post-filtration emulsion separation effluent, and water containing CFM-UiO-66-(COOH)_2_ for 30 days. The zebrafish were fed every 3 days with concurrent tank maintenance, while fish viability was recorded at 5 days intervals. As shown in Figure S6, no zebrafish mortality was observed in any of the experimental groups throughout the 30-day exposure period. Moreover, the zebrafish maintained high viability after 30 days of exposure. These results suggested that CFM-UiO-66-(COOH)_2_ exhibited good biosafety and biocompatibility with aquatic organisms, indicating its potential for practical applications in water treatment systems.

### 3.4. Demulsification and Separation Mechanism of CFM-UiO-66-(COOH)_2_

**Figure 6:**
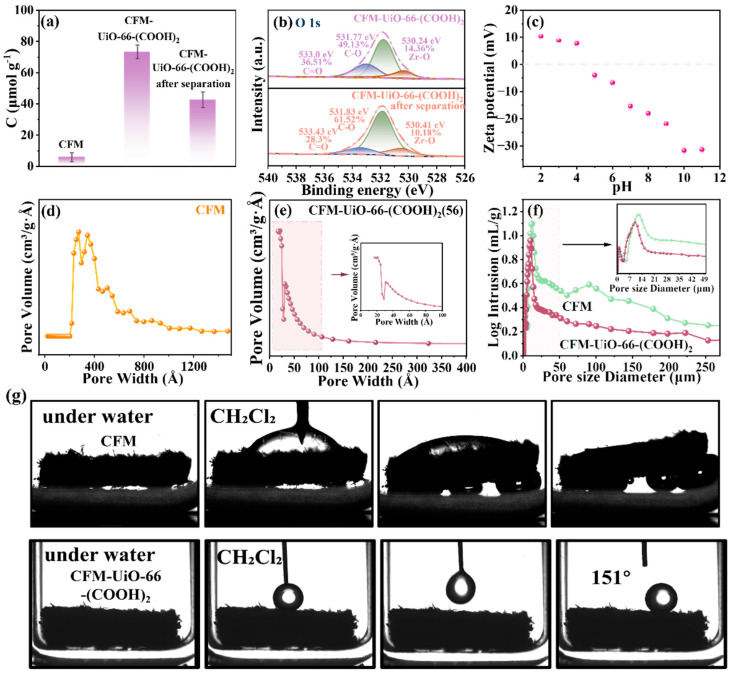
(**a**) Surface charge density of CFM and CFM-UiO-66-(COOH)_2_ before and after separation, (**b**) N 1s spectra of the CFM-UiO-66-(COOH)_2_ and CFM-UiO-66-(COOH)_2_ after separation, (**c**) The Zeta potential of CFM-UiO-66-(COOH)_2_ membrane, (**d**–**f**) The pore width of CFM and CFM-UiO-66-(COOH)_2_ and the mercuric depressor pore size distributions of CFM and CFM-UiO-66-(COOH)_2_, (**g**) The contact angle diagram of CFM and CFM-UiO-66-(COOH)_2_.

To investigate the emulsion separation mechanism of CFM-UiO-66-(COOH)_2_, colloidal titration was employed to quantify the change in anionic group concentration before and after separation. As shown in Figure 6a, the cationic group concentration decreased from 73.437 µmol·g^−1^ to 42.683 µmol·g^−1^ following NE1 emulsion separation, indicating the active involvement of anionic groups in the demulsification process. This conclusion was further supported by XPS analysis of the membranes before and after separation (Figure 6b). Specifically, post-separation XPS analysis of CFM-UiO-66-(COOH)_2_ revealed significant changes in the C=O bond characteristics: the peak intensity at 533.0 eV decreased from 36.51% to 28.3%, accompanied by 0.43 eV binding energy shift. These observations provided strong evidence for electrostatic neutralization between the positively charged cationic emulsion and the anionic CFM-UiO-66-(COOH)_2_. Zeta potential measurements confirmed that CFM-UiO-66-(COOH)_2_ remained negatively charged across a broad pH range (5–13) in aqueous solution. This stable surface charge contributed to the robust separation performance of the membrane under varying pH conditions, demonstrating excellent resistance to acidic and alkaline environments (Figure 6c).

Furthermore, to investigate the formation and size sieving effects of the selective layer in CFM-UiO-66-(COOH)_2_, detailed characterization was performed using BET analysis and high-pressure mercury intrusion porosimetry. Comparative pore size distribution analysis (Figure 6d–f) revealed a significant reduction in the average pore diameter of the membrane (1.590 nm) compared to pristine CFM (45.117 nm). This dramatic decrease in pore size provided direct evidence for the successful in situ growth of UiO-66-(COOH)_2_ on the collagen fiber membrane, effectively converting the original macroporous structure to a mesoporous structure. High-pressure mercury porosimetry further confirmed this structural transformation, revealing a distinct shift towards smaller pore sizes and a substantial reduction in macropore population. SEM characterization (Figure S7) clearly revealed that UiO-66-(COOH)_2_ crystals were uniformly grown throughout the CFM matrix, forming a dense, continuous MOFs selective layer firmly anchored to the collagen fibers. This well-integrated MOFs layer effectively blocks the passage of small emulsion droplets through size exclusion. Elemental mapping analysis revealed uniform distribution of C, N, O, and Zr across the membrane cross-section, confirming the homogeneous incorporation of UiO-66-(COOH)_2_ particles throughout the matrix and further supporting the formation of a continuous, sieving layer within the membrane structure.

The separation mechanism is further enhanced by the underwater oleophobicity of CFM-UiO-66-(COOH)_2_, which prevents oil droplet penetration while selectively allowing water molecules to pass, as demonstrated by its underwater oil contact angle measurements (Figure 6g and Figure S8). While unmodified CFM rapidly absorbs dichloromethane in aqueous environments, the CFM-UiO-66-(COOH)_2_ exhibited exceptional underwater superoleophobicity, maintaining an oil contact angle of 151°. This dramatic transition confirmed the successful modification of surface wettability through UiO-66-(COOH)_2_ integration. The formation of a superwetting interface facilitated complete hydration of CFM-UiO-66-(COOH)_2_, creating an oil-repellent surface that prevented emulsion droplet penetration. This combination of selective water permeation and simultaneous oil rejection serves as the key mechanism for achieving complete emulsion separation.

Thus, the separation mechanism of cationic O/W emulsions by membranes primarily involves three steps: (1) When cationic surfactant-stabilized emulsion droplets contact the membrane’s hierarchical micro-nanofiber surface, charge neutralization between the cationic surfactants and anionic carboxyl groups on the membrane promotes droplet deformation and spreading. (2) The migration of surfactants destabilizes the emulsion, causing droplet aggregation. (3) Finally, the aggregated droplets were rejected by the size-sieving layer formed by the UiO-66-(COOH)_2_ framework within the membrane, preventing their permeation and enabling efficient emulsion separation.

## 4. Conclusions

In summary, we have successfully developed a novel CFM-UiO-66-(COOH)_2_ for the efficient separation of cationic surfactant-stabilized emulsions. This membrane leveraged the inherent mechanical strength and capillary flow properties of a collagen fiber matrix substrate, combined with the tunability of in situ grown, functionalized UiO-66-(COOH)_2_. By precisely controlling the metal ion precursor concentration, the surface charge density and pore structure of the CFM were precisely engineered, resulting in a negatively charged membrane exhibiting high performance. The demulsification mechanism relies on a synergistic combination of charge neutralization and size sieving effects. The resulting composite membrane material achieved excellent separation efficiency (>99.85%) for various O/W emulsions stabilized by cationic surfactants, with a separation flux ranging from 178.9 to 225.9 L m^−2^ h^−1^. After six separation cycles, the separation flux of the membrane was in the range of 179.010 to 796.020 L m^−2^ h^−1^. Furthermore, the membrane demonstrated robust antifouling properties, excellent acid and alkali resistance (pH 1–13), high mechanical durability, and excellent biocompatibility. Moreover, a comparison of the separation performances for surfactant-stabilized emulsions with other reports is shown in Table S1. This is because the composite membrane material combines the excellent size-sieving capability of the leather collagen fiber membrane and the advantage of the functional sites of MOFs. As observed, the CFM-UiO-66-(COOH)_2_ showed excellent separation flux and efficiency relative to other membranes under lower pressures. These characteristics highlight the potential of the CFM-UiO-66-(COOH)_2_ as a durable, efficient, and environmentally responsible solution for complex emulsion separation challenges in a variety of industrial applications.

## Data Availability

The original contributions presented in this study are included in the article and Supplementary Materials. Further inquiries can be directed to the corresponding authors.

## Supplementary Materials

The following supporting information can be downloaded at: https://www.mdpi.com/article/10.3390/polym17212879/s1, Figure S1: The effect of membrane loading on emulsion filtration performance; Figure S2: XRD analysis of membranes under varying loading conditions; Figure S3: Stereomicroscopy images of ME emulsions with different compositions before and after filtration; Figure S4: DLS particle size distribution of NE; Figure S5: Digital images of CFM-UiO-66-(COOH)_2_ at different wear cycles; Figure S6: SEM images of CFM-UiO-66-(COOH)_2_ at different wear cycles; Figure S7: Survival rates of zebrafish in pure water, filtrate, and CFM-UiO-66-(COOH)_2_ containing water; Figure S8: Cross-sectional SEM image and corresponding elemental mapping of CFM-UiO-66-(COOH)_2_ containing water; Figure S9: Water contact angle (WCA) of the samples; Table S1: Comparison of the adsorption performance with other reported adsorbents. References [32,33,34,35,36] have been cited in supplementary materials.
