# Supplementary material for: UiO-66-(COOH)2 Decorated Collagen Fiber Membranes for High-Efficiency Separation of Cationic Surfactant-Stabilized Oil/Water Emulsions: Toward Sustainable and Robust Wastewater Treatment
Source: Polymers (Basel). 2025 Oct 29;17(21):2879. doi: 10.3390/polym17212879 (PMC12608758; doi:10.3390/polym17212879)
Supplement: Supplementary file 1 [file polymers-17-02879-s001.zip › polymers-3913072-supplementary.pdf]

Supplementary Information

Article

UiO-66-$(COOH)_2$ Decorated Collagen Fiber Membranes for High-Efficiency Separation of Cationic Surfactant-Stabilized Oil/Water Emulsions: Toward Sustainable and Robust Wastewater Treatment

Guifang Yang [1,2], Qiu Wu [3], Gao Xiao [3,*] and Xiaoxia Ye [3,*]

1 Fujian Provincial Key Laboratory of Ecological Impacts and Treatment Technologies for Emerging Contaminants, College of Environmental and Biological Engineering, Putian University, Putian 351100, China; abc396550322@ptu.edu.cn

2 Key Laboratory of Ecological Environment and Information Atlas, Fujian Provincial University (Putian University), Putian 351100, China

3 College of Environment and Safety Engineering, Fuzhou University, Fuzhou 350108, China; 230620061@fzu.edu.cn

* Correspondence: xiaogao@fzu.edu.cn (G.X.); yexiaoxia@fzu.edu.cn (X.Y.)

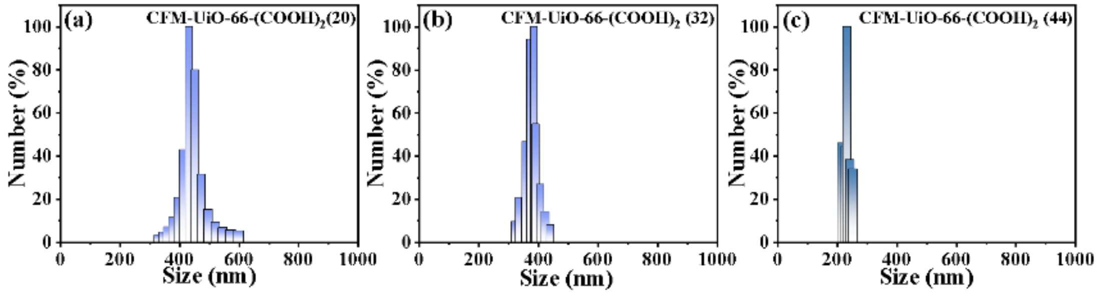

Fig. S1 The effect of membrane loading on emulsion filtration performance.

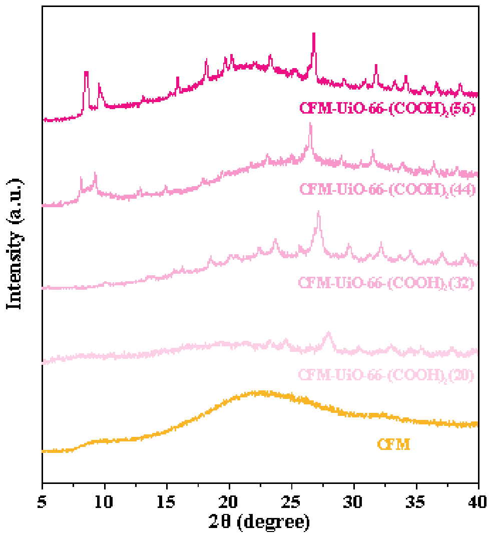

Fig. S2 XRD analysis of membranes under varying loading conditions.

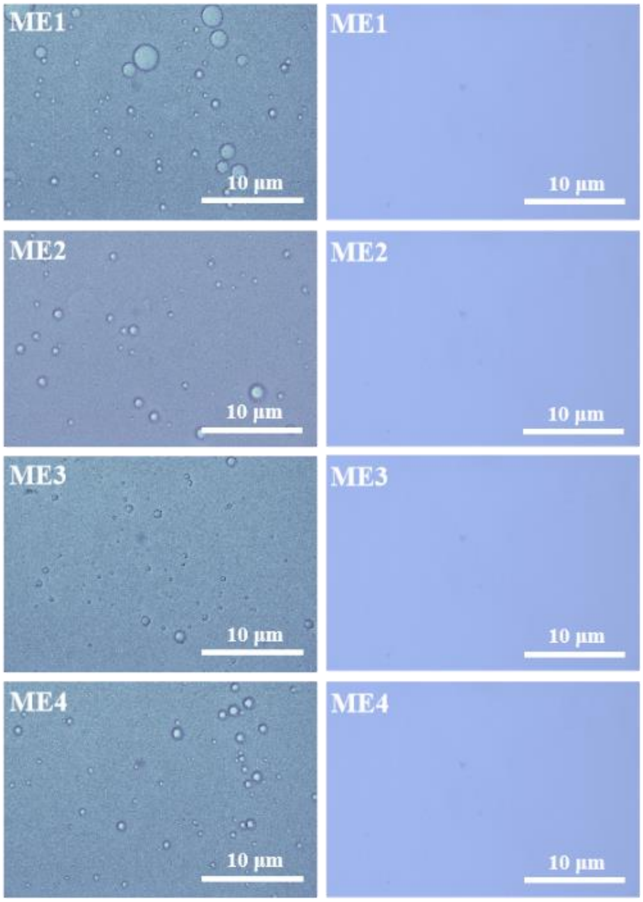

Fig. S3 Stereomicroscopy images of ME emulsions with different compositions before and after filtration.

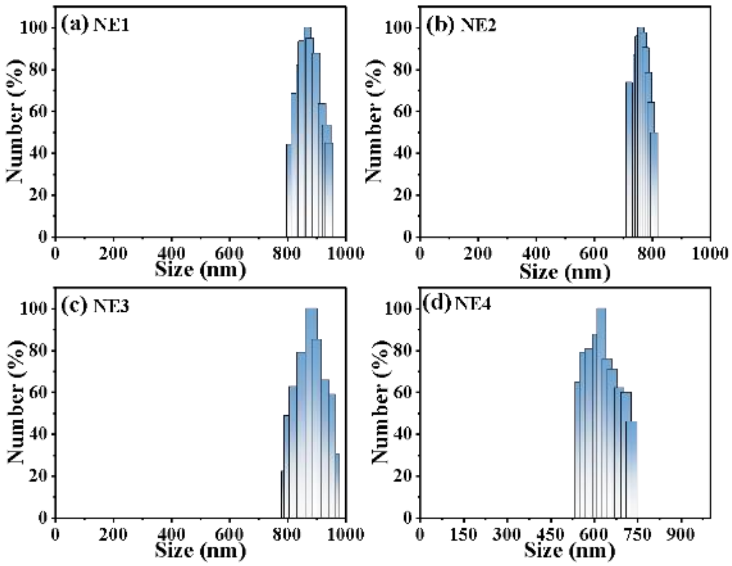

Fig. S4 DLS particle size distribution of NE.

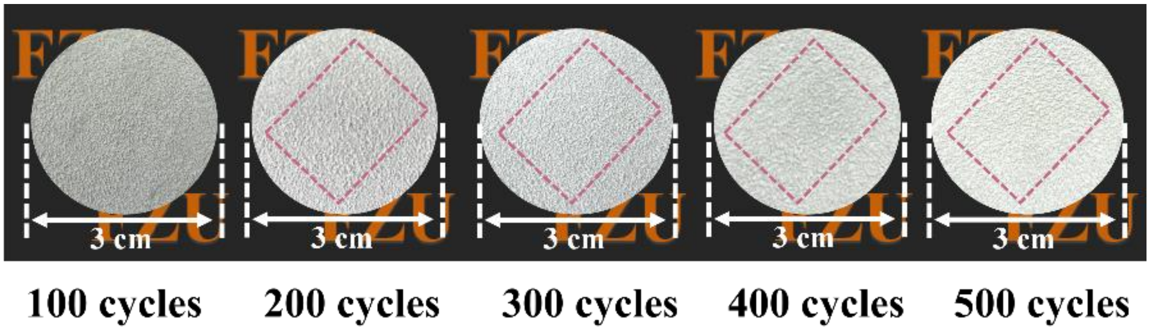

Fig. S5 Digital images of CFM-UiO-66-$(COOH)_2$ at different wear cycles.

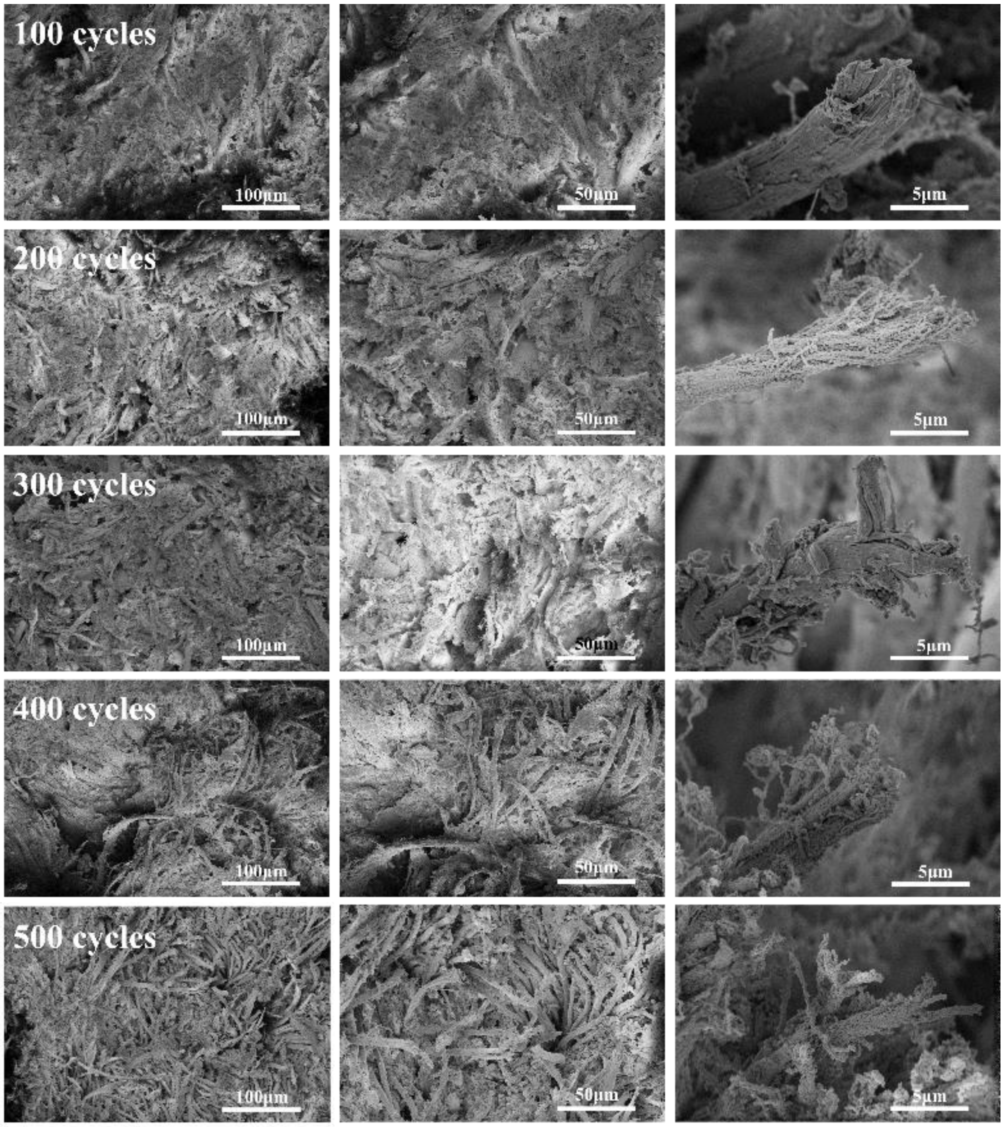

Fig. S6 SEM images of CFM-UiO-66-$(COOH)_2$ at different wear cycles.

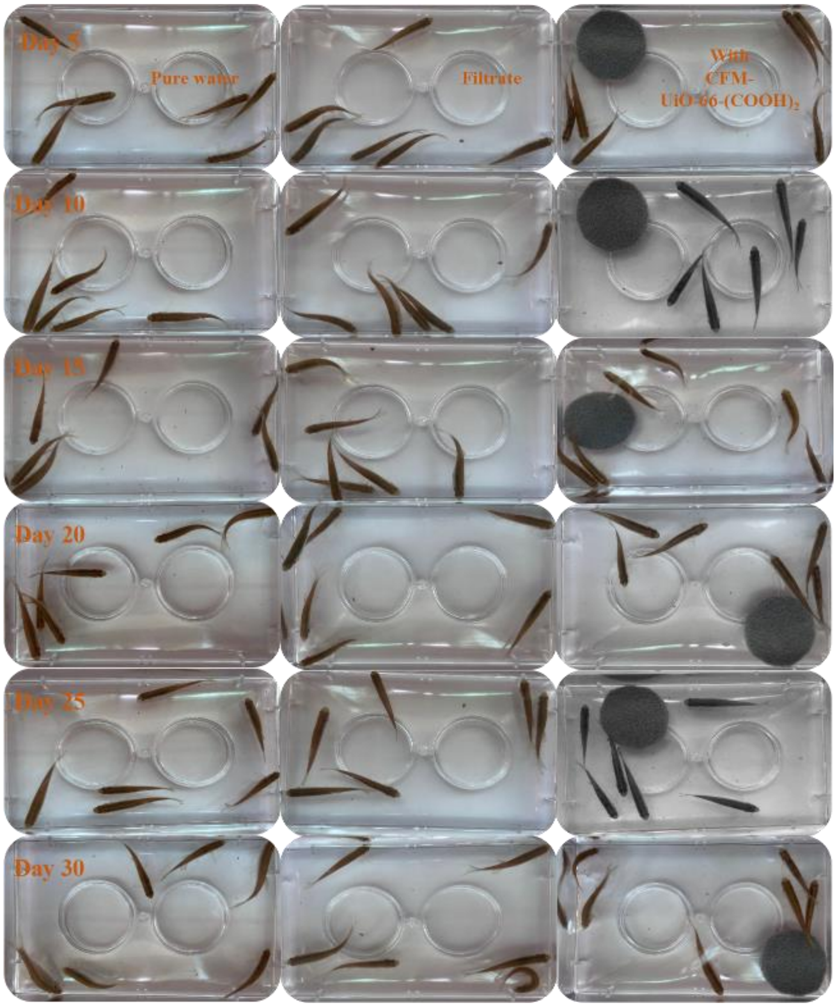

Fig. S7 Survival rates of zebrafish in pure water, filtrate, and CFM-UiO-66-$(COOH)_2$ containing water.

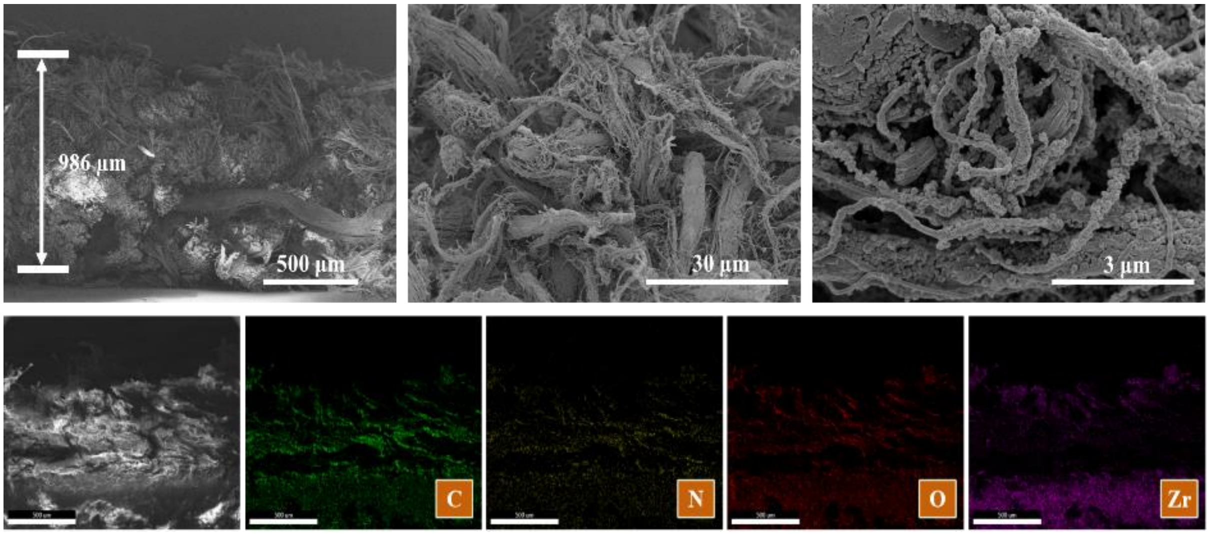

Fig. S8 Cross-sectional SEM image and corresponding elemental mapping of CFM-UiO-66-$(COOH)_2$.

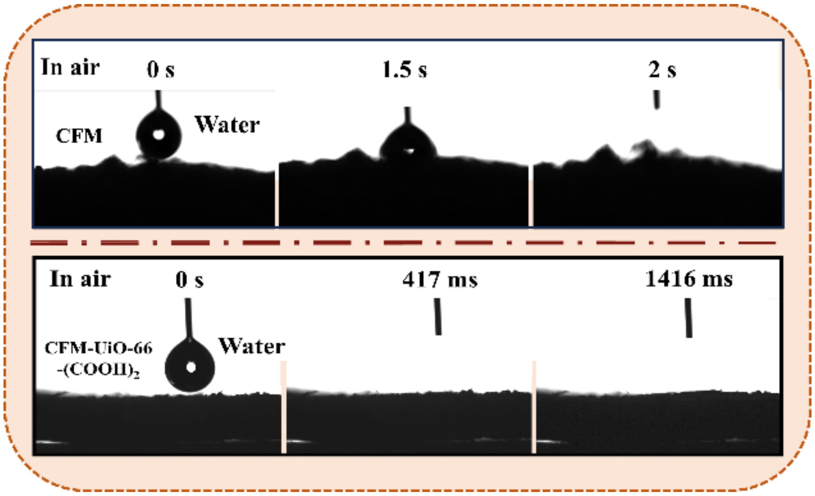

Fig. S9 Water contact angle (WCA) of the samples.

Table. S1 Comparison of the adsorption performance with other reported adsorbents.

Membrane materials	Flux ($L\ m^{-2}\ h^{-1}$)	Separation efficiency (%)	Driving force (MPa)	Ref.
TA-Ti@PVDF	689.6	99.35	0.01	(Wu et al., 2021)
TA-APTES-Fe^{3+}@PEG-SiO_2-PVDF (TP-7)	678	99.57	0.01	(Zhong et al., 2024)
ZIF-8/P(AN-MA)	796-3053	>97.5	/	(Fan et al., 2024)
MXene@UIO-66-$(COOH)_2$	498.91±38.75	>99.26	0.09	(He et al., 2020)
TA/EDTA-2Na/PVDF	754.4	99.51	0.09	(Gao et al., 2021)
CFM-UiO-66-$(COOH)_2$	179.010-796.020	99.851	gravity	This work

References

Fan F, Zhao L, Guo Y, Xu H, Wang T, Fu Y (2024). Fabrication of a ZIF-8/PVA Membrane on PVDF Fiber by Spray for the Highly Efficient Separation of Oil-in-Water Emulsions. ACS Applied Materials & Interfaces, 16(40): 54548-54554

Gao J, Cai M, Nie Z, Zhang J, Chen Y (2021). Superwetting PVDF membrane prepared by in situ extraction of metal ions for highly efficient oil/water mixture and emulsion separation. Separation and Purification Technology, 275: 119174

He S, Zhan Y, Hu J, Zhang G, Zhao S, Feng Q, Yang W (2020). Chemically stable two-dimensional MXene@UIO-66-$(COOH)_2$ composite lamellar membrane for multi-component pollutant-oil-water emulsion separation. Composites Part B: Engineering, 197: 108188

Wu J, Hou Z, Yu Z, Lang J, Cui J, Yang J, Dai J, Li C, Yan Y, Xie A (2021). Facile preparation of metal-polyphenol coordination complex coated PVDF membrane for oil/water emulsion separation. Separation and Purification Technology, 258: 118022

Zhong X, Shi Q, Guo Z (2024). Synergistic Construction of Superhydrophilic PVDF Membranes by Dual Modification Strategies for Efficient Emulsion Separation. Small, 20(37): 2402538
